# Low-dose mesenchymal stem cell therapy for discogenic pain: safety and efficacy results from a 1-year feasibility study

**DOI:** 10.2144/fsoa-2021-0155

**Published:** 2022-04-21

**Authors:** Dan Bates, David Vivian, Julien Freitag, James Wickham, Bruce Mitchell, Paul Verrills, Kiran Shah, Richard Boyd, Dean Federman, Adele Barnard, Lera O'Connor, Jacqui F Young

**Affiliations:** 1 Melbourne Stem Cell Centre, Box Hill, Victoria, 3128, Australia; 2 Magellan Stem Cells, Victoria, Australia; 3 Metro Pain Group, Clayton, Victoria, 3168, Australia; 4 Monash Clinical Research, Clayton, Victoria, 3168, Australia; 5 School of Dentistry & Medical Sciences, Charles Sturt University, Wagga Wagga, NSW, 2650, Australia; 6 The Hudson Institute, Clayton, Victoria, 3168, Australia; 7 Capital Radiology, Clayton, Victoria, 3168, Australia

**Keywords:** chronic pain, disc degeneration, discogenic low back pain, feasibility study, mesenchymal stem cells

## Abstract

**Aim:**

To evaluate safety and efficacy of low dose autologous adipose-derived mesenchymal stem cells (ADMSCs) for treatment of disc degeneration resulting in low back pain (LBP).

**Methods:**

Nine participants with chronic LBP originating from single-level lumbar disc disease underwent intradiscal injection of 10 million ADMSCs with optional repetition after 6 months.

**Results:**

No unexpected or serious adverse events were recorded. Seven (78%) of participants reported reductions in pain 12 months after treatment. Five (56%) reported increased work capacity. Three (33%) reduced analgesic medication. Improvements in EQ-5D and Oswestry disability index results were observed. MRI demonstrated no further disc degeneration and improvements to annular fissures and disc protrusions.

**Conclusion:**

This study provides initial evidence of safety and efficacy of ADMSCs for discogenic LBP.

## Plain language summary

This feasibility study sought to evaluate the safety and efficacy of low dose autologous adipose-derived mesenchymal stem cells (ADMSCs) for treatment of disc degeneration resulting in low back pain (LBP) in 9 participants. No unexpected or serious adverse events were recorded. Seven (78%) of participants reported reductions in pain 12 months after treatment. Five (56%) reported increased work capacity. Three reduced analgesic medication. Quality of life improvements were also observed. **Conclusion:** This study provides initial evidence of safety and efficacy of ADMSCs for discogenic LBP.

## Background

Low back pain (LBP) is a major health problem, affecting approximately 60–80% of the adult population at some stage in their life [1–3]. LBP is the second most common reason for physician visits [1] and work disability [4] and is associated with substantial healthcare costs and work absenteeism [5–7]. In Australia, the most frequently seen musculoskeletal conditions in general practice are associated with back problems, with approximately 11% of the population experiencing high-disability LBP [3,8,9]. The intervertebral disc (IVD) is a common source of LBP, being the prime source in about 40% of complex chronic LBP presentations and discogenic LBP often has a poor prognosis [10,11].

The etiology of degenerative disc disease (DDD) is well established. The vertebral endplate is subject to fatigue failure [12–14] causing de-aggregation of proteoglycans in the nucleus, a reduction in water content and non-collagenous proteins, consequent depressurization of the nucleus pulposus and delamination of the annulus fibrosus [15]. The pathology is also characterized by secondary marginal vertebral body osteophyte formation, possible shrinkage of the nucleus pulposus and prolapse or folding of the annulus.

The hallmarks of DDD are reduced disc height and disc desiccation. The prevalence and progression of disc height reduction over 12 months has been reported in a number of papers with varying populations. Still, it has been shown that it occurs more frequently in the lumbar spine, is more common in women than in men [16], and degeneration can increase depending on disc position with degeneration increasing as one progresses caudally down the spinal column [17] and disc height reduction being reported at levels of 3–15%/year [18–20].

Pain as a result of disc degeneration/pathology commonly presents as central low back pain though may be associated with referral to surrounding tissues or even the lower limb with this pain reportedly resulting from aberrant healing responses within the structure of the disc and the disc becoming densely innervated [21]. Discogenic pain is typically confirmed when symptoms are reproduced/‘provoked’ in provocation discography [22,23].

Treatment options in the past have centered primarily around pharmacological and/or surgical interventions. Non-invasive options such as physical therapy and pain management programs have limited evidence for success for LBP caused by DDD. Further, surgical interventions such as discectomy and fusion often yield uncompelling outcomes and may come with significant morbidity [24].

Concerns regarding traditional management have led to the exploration of minimally invasive cell therapy strategies that can putatively regenerate the IVD and restore or improve its function [25,26]. Cell therapies typically offer an attractive safety profile and have lower costs compared to surgery. Mesenchymal stem cells (MSCs) have been shown to be a promising cell source for use in restoring the normal cellular constitution of the degenerated disc and they are also readily available in a number of tissues (e.g., adipose, bone marrow or umbilical cord blood).

The ability of MSCs to differentiate along a mesodermal lineage initially led to interest in their potential role in assisting tissue repair [27–29]. New hypotheses indicate that MSCs regenerate injured tissues via their cell-to-cell interaction and paracrine secretions that lead to the stimulation of trophic pathways [30–32], a reduction in cell apoptosis, enhancement of cell proliferation and inhibition of inflammatory mechanisms including immunomodulatory pathways. Specifically, the alternative modes of repair by MSCs include paracrine activity of secreted growth factors, anabolic cytokines including TGF-β, VEGF and EGF and hormones. In addition, the cell-to-cell interactions are enhanced and mediated by tunneling nanotubes, and release of extracellular vesicles (including exosomes) that contain reparative peptides/proteins, mRNA and microRNAs [33]. It is this observed paracrine expression and cell–cell interaction that is now considered their primary mechanism of action and likely role in tissue repair rather than via direct differentiation [30,31,34,35].

A recent pre-clinical animal study showed that implantation of bone marrow-derived MSCs to degenerative discs inhibited fibrosis/scarring, thereby preserving mechanical properties and overall spinal function [36]. Furthermore, studies of participants with confirmed disc-related LBP and injected with autologous bone marrow-derived MSCs, have described improvements in both pain and disability up to 24 months after injection [37–41].

With continued interest in the possible clinical applications of MSC therapies it is imperative to determine not just efficacy but also safety. Based upon current clinical trial outcomes, MSC therapy is relatively low-risk. A recent meta-analysis of 36 trials involving a total of 1012 participants receiving MSC therapy for various conditions, did not identify any significant adverse events other than transient and self-limiting fever [42]. Further, a systematic review of clinical studies involving the use of intra-articular injections of autologous expanded MSCs with a mean follow-up of 21 months of 844 procedures showed no association with adverse events such as infection, death or malignancy [43].

Preliminary research on MSCs was performed using bone marrow-derived cells. However, bone marrow harvest procedures are painful and yield low numbers of MSCs [44]. An alternative source of autologous adult MSCs, due to its abundance and ease of harvest, is adipose tissue [45–47]. Also, autologous adipose-derived mesenchymal stem cells (ADMSCs) retain their inherent stem cell properties (i.e., ability to self-renew and differentiate) as well as their multi-potency and immunomodulatory properties following prolonged culture expansion [48–51].

The results of previous pre-clinical and clinical trials using bone marrow-derived MSCs are encouraging and support the potential use of ADMSCs in the treatment of disc-related LBP with the ability to reduce pain and assist in intervertebral disc regeneration [52]. This treatment has the potential to significantly reduce disability from LBP, also leading to significant economic benefits. Kumar *et al.* have previously reported promising results with ADMSCs at high cell concentrations utilizing hyaluronic acid (HA) as carrier for the ADMSCs [52]. As the authors note, HA can also contribute to the efficacy recorded [52]. Here, we evaluate the safety and efficacy of low dose ADMSC therapy for LBP and utilize an isotonic solution as carrier, rather than HA, to ensure the measured outcomes could be confidently attributed to the efficacy of the ADMSCs alone.

## Materials & methods

### Study design

This primary objective of this prospective, single-center, feasibility study was to assess the safety and efficacy of low dose ADMSCs in participants with single level disc degeneration with symptomatic discogenic LBP.

The secondary objective was to assess the potential of low dose ADMSC therapy to achieve positive quality of life outcomes and disease modification by reducing the progression of DDD or regeneration of the IVD. MRI was used to assess putative morphological changes in the IVD.

**Figure 1.**
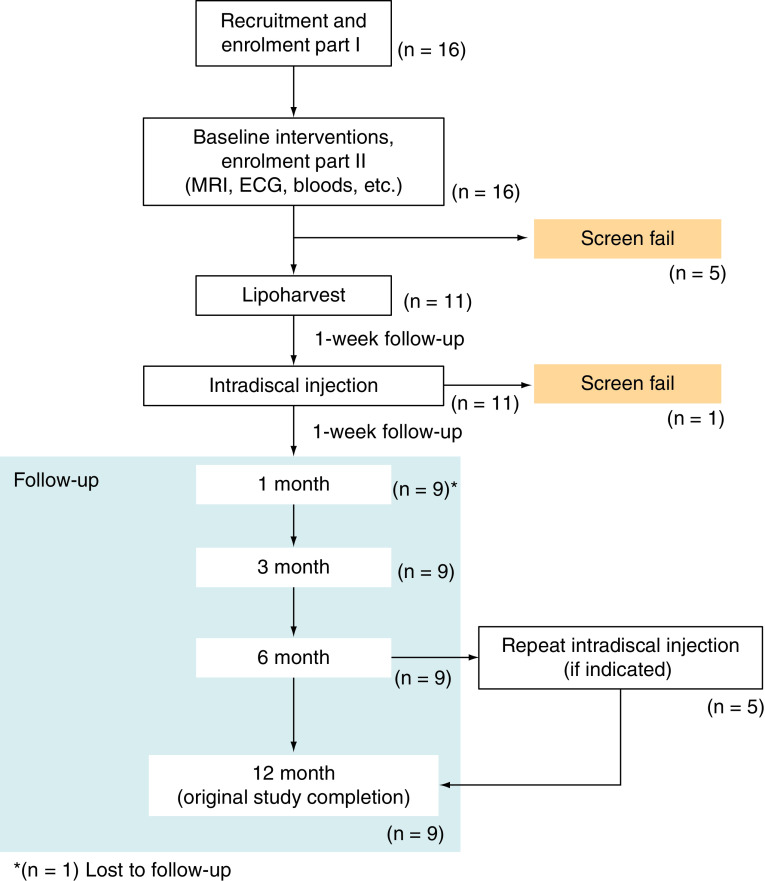
A flow chart highlighting the study design of this trial.

An outline of the study design together with the number of participants involved at each timepoint is shown below (Figure 1). Following ADMSC therapy, participants attended the clinic and received phone calls to complete the primary safety and efficacy outcome measures at 1, 3, 6, 9 and 12-months post-treatment. Unscheduled visits were also allowed at any time for safety reasons or for the assessment of any adverse events as required.

#### Participants

Participants were enrolled in the study if they met all the eligibility criteria outlined in Table 1.

**Table 1.** Eligibility criteria for participants.

Key inclusion criteria
1. Chronic LBP of discogenic origin for at least six months
2. LBP ≥ 5/10 on numeric pain rating scale at enrolment
3. Aged 18 years or older who have previously had three months of unsuccessful conservative back pain care, including, for example, medications (anti-inflammatories, analgesics, muscle relaxants), massage, acupuncture, chiropractic manipulations, activity modification, home directed lumbar exercise programs
4. If present, leg pain <LBP, and of nociceptive not radicular type
5. MRI findings: a) One level disc degradation without disc space height loss of more than 50% +/- Modic type I or II changes at the same level b) May include contained disc herniations ≤3 mm protrusion with no imaging evidence of neurological compression

Key exclusion criteria
1. Pregnancy or breast feeding (accepted contra-indication as no safety data on this population).
2. Discogram positive patients with a grade 5 annular tear (it is not known what the effect of dilution will have when the cells penetrate through the annulus fibrosus into the epidural space)
3. A prior history of cancer or other malignant condition, or an atypical chronic pain syndrome
4. A history of epidural steroid injections within eight weeks prior to treatment injection
5. Previous surgery at the symptomatic disc that has altered the structure of the target disc level (e.g. discectomy, laminectomy, foraminotomy, fusion, intradiscal electrothermal therapy, intradiscal radiofrequency, thermocoagulation)
6. A current diagnosis of bleeding disorders and/or taking prescribed anti-coagulants that cannot be ceased
7. A history of allergy to any substances used within the treatments

LBP: Low back pain.

As part of the study, safety requirements to determine the health of participants before any procedures were administered, including a standard 12-lead ECG, a full blood count, liver and renal function tests with clotting time assessment. Females of childbearing potential underwent a pregnancy test.

#### Autologous MSC production method: harvesting

Following the baseline visit, MRI and confirmation of eligibility criteria, participants underwent the abdominal lipoharvest procedure.

The methods of harvesting, isolation and expansion of autologous MSCs, which have previously been published were undertaken in this study [53]. In summary, an abdominal liposuction was performed under local anesthetic tumescence control with a total of 40–100 ml of lipoaspirate collected using a manual syringe suction technique or via mechanical suction with collection in a sterile single-use filtered container (Shippert Medical, CO, USA). Participants received a single dose of IV antibiotics at the time of the lipoharvest procedure as part of accepted routine infection control prophylaxis [54].

All participants were reviewed the week after the cell harvest procedure by the treating doctor to assess for wound healing and any adverse events.

#### Autologous MSC production method: isolation & expansion

The lipoaspirate was transferred directly after harvest to an onsite clean room tissue culture laboratory and processed in a sterile environment in a Biological Safety Cabinet (BSC) class II using strict aseptic techniques. The cleanroom was graded as ISO5 air quality or greater. All the equipment and solutions used were qualified and validated for aseptic use in cell culture, including all reagents and buffer. The cells were then expanded in sterile tissue culture conditions and dosages containing approximately 10 million autologous MSCs each were frozen individually in sterile cryovials in approved cell-safe cryoprotectant media by a validated control rate freezing technique and stored in liquid nitrogen until required [55,56]. The formal isolation and expansion protocol has been previously published [53].

Cells were characterized by flow cytometry (FACS) following previously published standards of the International Society for Cellular Therapy [57]. Samples were assessed for the presence of MSC specific surface antigens (CD90, CD73 and CD105) and the absence of hemopoietic surface markers (CD14, CD19, CD34 and CD45). The cultured cells were also tested for microbial and fungal contamination by culturing a sample of each patient's cells for 7 days at a qualified independent microbiology laboratory with negative growth result.

On the day of treatment, a single dose of cells was thawed at 37°C in a sterile waterbath, repeatedly washed with chilled phosphate buffered saline (PBS) and centrifuged to remove the cryoprotectant media. The pelleted cells were then resuspended and prepared for injection by mixing with 1 ml of sterile clinical-grade isotonic solution (Plasma-Lyte 148).

Prior to transfer to the procedure center for injection, cell number and viability was assessed using a Muse Cell Analyzer (Merck, Millipore, USA) (Table 2). In-house assessment has shown stem cell number and viability to be stable for 24 h after preparation when stored at 2–8°C until use. Intra-discal injection was performed within this time period.

**Table 2.** Average cell count and viability confirmed using a muse cell analyzer.

	Baseline ADMSC injection (n = 9)		6-month ADMSC injection (n = 5)	
	Cell count (millions)	Cell viability (%)	Cell count (millions)	Cell viability (%)
Mean	10.9	96.6	10.2	96.8
SD	0.99	1.17	0.4	0.98

ADMSC: Adipose-derived mesenchymal stem cell; SD: Standard deviation.

#### Disc injection method

On day 0/treatment administration day, each patient received a 1 ml intradiscal injection of 10 million ADMSCs in injectable grade Plasma-Lyte 148. Under sterile conditions and live C-arm fluoroscopy, the intradiscal injection into the symptomatic disc was performed using a right postero-lateral approach. All injections were performed by the same physician to ensure standardization of technique.

Before this injection, all participants (with the exception of those who had previously had a discogram) underwent low pressure provocation discography according to the Spine Intervention Society standards [22] to reproduce their LBP and further confirm the treatment disc.

Treatment injection and post-procedure care (anesthesia, prophylactic antibiotics, post-procedure analgesia) were performed in accordance with standard of care as appropriate in the judgment of the treating physician. All participants were seen one-week post-injection to assess for any signs of infection and to evaluate the extent of any post-procedure pain flares.

### Outcome measures

The tools used to assess the primary and secondary outcome measures of formal function and pain comprised several validated questionnaires which are detailed below in Table 3.

**Table 3.** Tools used for the evaluation of outcome measures.

Outcome measures	Measurement point (months)
Primary outcome measure	
NPRS	0, 1, 3, 6, 9, 12

Secondary outcome measures	
DASS21	0, 1, 3, 6, 9, 12
ODI	0, 1, 3, 6, 9, 12
Standing, sitting and walking tolerances	0, 1, 3, 6, 9, 12
EQ-5D-3L	0, 1, 3, 6, 9, 12
Change to employment capability	0, 1, 3, 6, 9, 12
Patient satisfaction	1, 3, 6, 9, 12
PGIC	1, 3, 6, 9, 12
MRI	0, 12

DASS21: Depression, anxiety and stress scale; NPRS: Numeric pain rating scale; ODI: Oswestry disability index; PGIC: Patient global impression of change.

#### Primary outcome measures

For the numeric pain rating scale (NPRS), participants rated their average pain and most severe pain intensity over the past week on a scale of 0–10. The NPRS has been validated for use in people with LBP [58,59].

The participants were questioned for the occurrence of adverse events (AEs) during the study period. AEs were summarized by severity, treatment/intervention provided, relationship to the study procedures and resolution.

#### Secondary outcome measures

The patient global impression of change (PGIC) scale provides the opinion of the patient as to how they believe they have responded to treatment and is commonly used in conjunction with the NPRS to determine meaningful results [60–62]. The validated questionnaires DASS21 [63], ODI [64,65], EQ-5D-3L [66–68] were used in accordance with their instructions. The DASS21 questionnaire was provided to participants, and the results tallied then multiplied by two for scoring within the validated DASS42 scale. For the standing, sitting and walking tolerances, participants were asked to provide the number of minutes they could comfortably perform these activities before pain prevented them from continuing. In addition to questionnaires, all pain medications were recorded at the same timepoints.

By examining structural changes using MRI, it may be seen if ADMSC therapy offers disease-modifying potential, in other words, reducing progression of DDD. MRIs were performed at baseline and 12 months. MRI analysis was performed on a 1.5T or greater MRI system with a dedicated spinal coil. Sagittal T1, sagittal T2, axial T2 and coronal T2 fat saturation sequences were performed.

MRIs were analyzed focusing on modic changes, disc desiccation, disc height, annular tears and disc protrusions. MRIs were assessed by a specialist musculoskeletal radiologist.

#### Statistics

Continuous variables were reported as means and standard deviations, or percentage change where appropriate. Categorical variables were reported as counts and percentages where possible. Due to the small sample size, statistical significance calculations were not performed.

## Results

### Study subjects

A total of 16 subjects were screened at commencement of the trial with the intention of recruiting ten participants. five subjects failed screening and an additional single subject failed screening at time of treatment where it became evident that he suffered from two levels of disc degeneration rather than single level. A further single subject was lost to follow-up with a total of nine subjects completing follow-up.

To provide accurate data, the results presented, including baseline data, are based on 90% (n = 9) of the original study population, as one subject was lost to follow-up after the initial lipoharvest and stem cell injection, so had no comparable data available.

### Demographics

A summary of participant demographics is provided in Table 4.

**Table 4.** Participant demographics.

Gender, male vs female	56 vs 44
Age (years)	40.1 (10.3)
Height (m)	1.7 (0.1)
Weight (kg)	75.3 (17.6)
BMI (kg/m^2^)	25.4 (3.1)

Standard deviation in brackets.

Participants had been suffering from chronic LBP, with two participants (22%) experiencing pain for 1–2 years, four participants (44%) suffering from pain for 3–5 years and three participants (33%) suffering from pain for over 10 years.

### Employment

At baseline, six of the nine (67%) participants were working between 26 and 52 h per week.

After 12 months, working participants increased to seven of the nine (78%), with four participants (44%) showing improvement in their capacity to perform work, and two participants (22%) reporting no change to working capacity. One participant was previously unable to work but returned to full time work after 12 months. One participant recorded work as not applicable and one participant was unable to work for the duration of the study.

Three participants (33%) noted an increase in the number of hours they were able to work, with the most significant change seen in a participant who had previously been unable to work and returned to full-time employment. This participant increased the number of working hours over the 12-month period, recording 30 hours per week at 6 months and 38 hours at 12 months.

### Medications

Of the eight participants taking regular and *pro re nata* (PRN) medications at baseline to manage their pain condition, a reduction in medication was recorded in three (38%) of these, whilst another was able to cease all medication by the 12-month follow-up visit.

### Pain outcomes

#### Average pain

**Figure 2.**
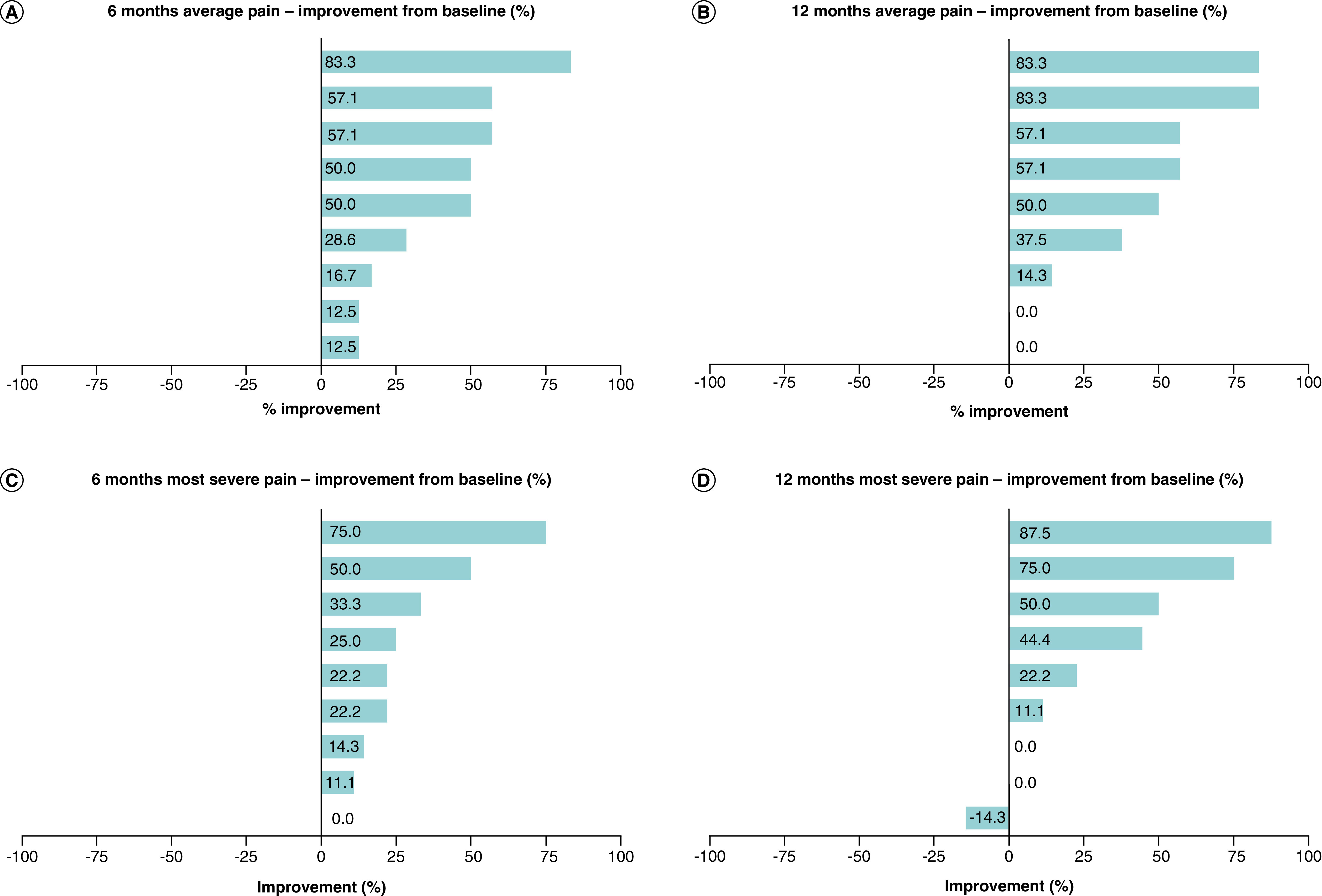
Tornado plots showing percentage improvement in numeric pain rating scale scores for each participant. **(A)** Average pain scores at 6 months. **(B)** Average pain scores at 12 months. **(C)** Most severe pain at 6 months. **(D)** Most severe pain at 12 months.

All participants reported an improvement in their average pain scores at 6 months following injection. At both 6 and 12 months, five of the nine participants (55%) reported improvements of ≥50% in their average pain score (Figure 2A & B). Two participants reverted to baseline average pain levels, one of which was in response to an unrelated work injury encountered just before the 12-month timepoint.

#### Most severe pain

Eight (89%) and six (67%) of the nine participants reported an improvement from baseline in their most severe NPRS score by 6 and 12 months, respectively (Figure 2C & D). Three participants (30%) demonstrated improvements of ≥50% at 12 months. The above-mentioned participant who experienced an unrelated work injury also reported a worsening of their most severe pain in comparison to baseline at the 12 month follow-up visit.

### Pain outcomes & number of cells injected

Participants were assessed at 6 months post-injection and given the option of a second injection, if they continued to experience ongoing intrusive pain. Following consultation with the physician, five participants (56%) underwent a second injection of a further 10 million ADMCSs at this point, totaling 20 million cells injected.

Of those who received a second injection (n = 5), at 12 months, 2 participants (40%) improved, 2 participants (40%) worsened and one participant (20%) showed no changes compared to their 6 month results in both average and most severe pain.

### Tolerance for standing, sitting & walking

**Figure 3.**
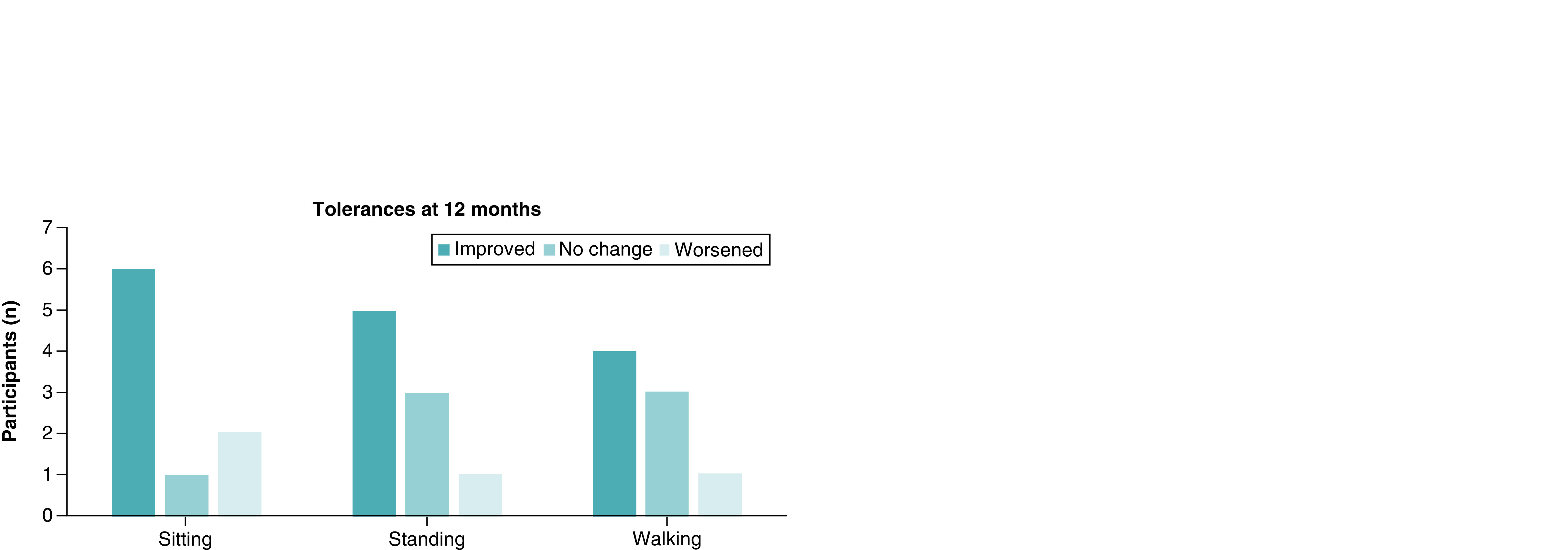
Changes to duration of standing, sitting and walking tolerance after 12 months.

Participants were asked to record how long they were able to sit, stand and walk for at each time interval, before the pain became unbearable. After 12 months post-procedure, six participants (67%) reported improvements in sitting tolerance duration, one participant (11%) reported no change and two participants (22%) reported worsening. In terms of standing tolerance, five participants (56%) reported improvements, three participants (33%) reported no change and one participant (11%) reported worsening at the 12-month time interval. One participant did not record a final entry for walking tolerance, indicating either a missed question or no restriction to his walking ability (Figure 3). Among the participants who provided data, four (50%) reported improvements in walking tolerance duration, three participants (38%) reported no change and one participant (13%) reported worsening at the 12-month time interval. For participants who improved at 12 months, the median observed treatment effect was at least 1.5 times the baseline capacity (2.0 × [sitting] 1.5 × [standing] and 1.75 × [walking]).

### Functional outcomes

**Figure 4.**
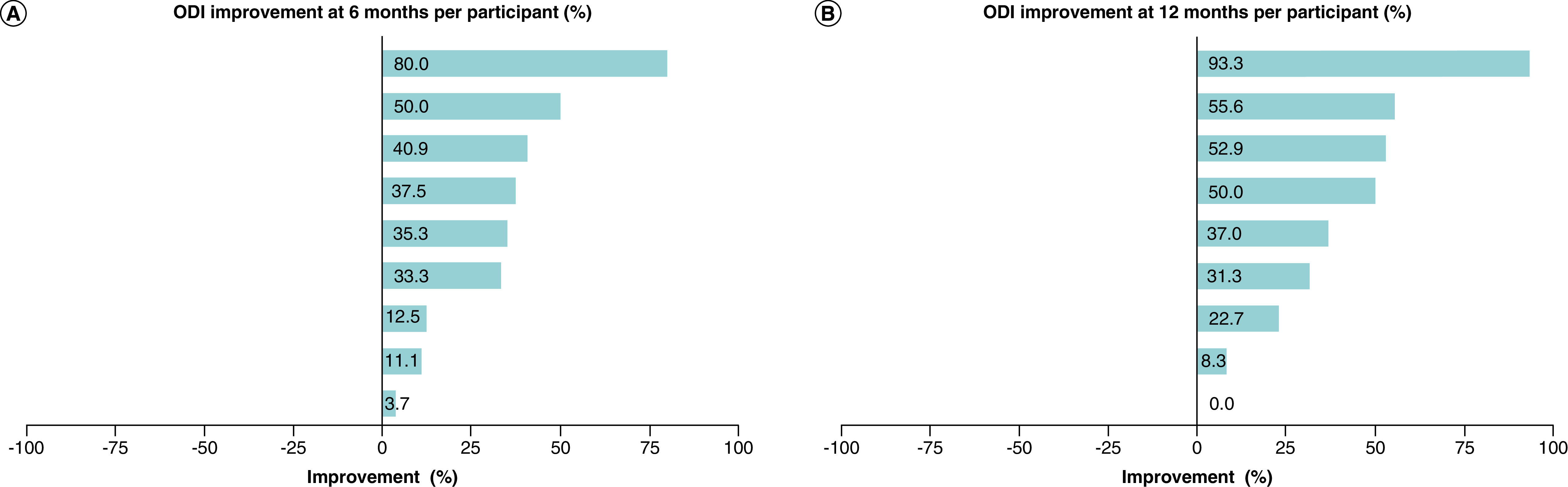
ODI results showing percentage improvement in functional outcomes for each participant. **(A)** 6 months. **(B)** 12 months.

The majority of participants showed improvements in functional outcome scores at 6 and 12 months as measured by both the ODI and EQ-5D-3L. At 6 months, all participants had recorded an improvement in functional outcomes in the ODI compared to baseline, with improvements ranging from 4 to 80% (mean: 34%) (Figure 4A). At 12 months, 89% (n = 8) of participants recorded improvements from baseline ranging from 8 to 93%, while one participant (11%) reverted to their original baseline value (mean: 39%) (Figure 4B). At both time intervals, 67% of participants (n = 6) reported improvements ≥30%.

**Figure 5.**
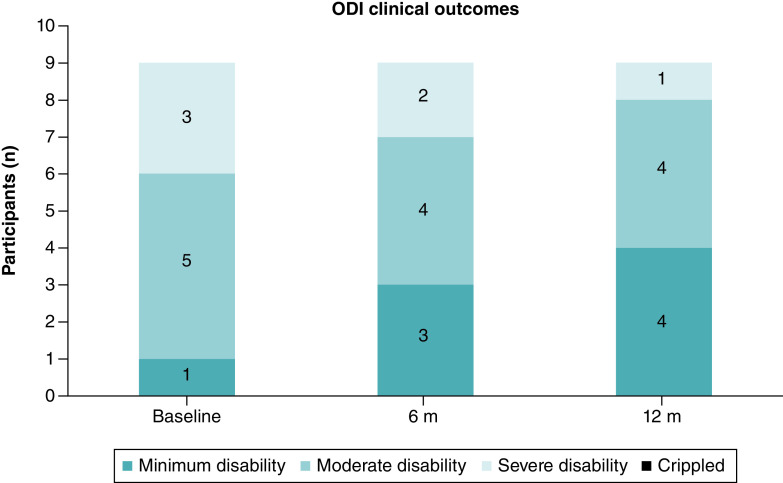
ODI results showing clinical outcomes at baseline, 6 and 12 months.

At 12 months, 5 participants (56%) had shifted into milder clinical categories within the ODI (two moved from severe-to-moderate disability, and three moved from moderate to minimum disability) (Figure 5).

**Figure 6.**
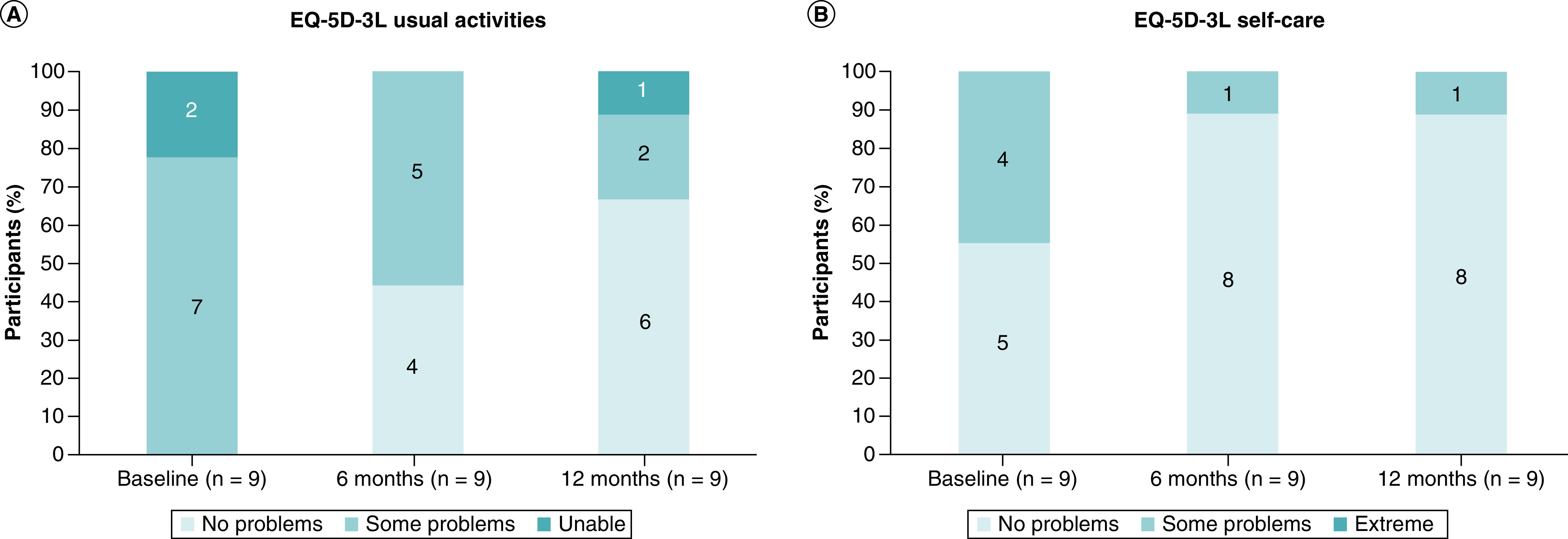
Percentage of participants reporting each category in functional outcomes (EQ-5D-3L) at baseline, 6 and 12 months. **(A)** Usual activities. **(B)** Self-care.

As measured by the EQ-5D-3L, all participants reported some difficulties with undertaking their usual activities (work, study, housework, family or leisure activities) at baseline (Figure 6A). Encouragingly, after 6 months, 44% (n = 4) of participants improved to report no difficulties, which further increased to 66% (n = 6) after 12 months. In addition, one participant (11%) was previously unable to perform their usual activities at baseline and improved to only having some problems. One participant (11%) showed improvement at 6 months but reverted to baseline scores after 12 months, and one participant did not show any changes throughout the study.

While four participants (44%) noted some problems with self-care (washing and dressing) at baseline, the only participant who had problems with self-care after 12 months was the participant who had suffered an unrelated work injury just before the 12-month timepoint (Figure 6B). This participant's earlier results at 6 months showed no problems with self-care.

The other measures of the EQ-5D-3L (including mobility, pain/discomfort and anxiety/depression) saw improvements for one to two participants per measure, while the other participants were stable and did not record any worsening.

### DASS results

It is worth noting that baseline DASS scores showed that the majority of participants (67%; n = 6) recorded normal scores in all three domains of depression, stress and anxiety. In all subcategories, 67% (n = 6) recorded normal scores at 12-month follow-up.

From baseline to 12 months, a slight improvement was observed in the stress scale (median overall improvement of 2 units across all participants, individual changes ranged from -6 to +6 units) while no consequential change was observed in the anxiety scale (median overall change of 0 across all participants, individual changes ranged from -6 to +6 units).

The depression scale varied more, with individual scores ranging from -8 (deterioration) to +18 (improvement) between baseline and 12 months, the median change across the scores being 2.

### Patient Global Impression of Change

**Figure 7.**
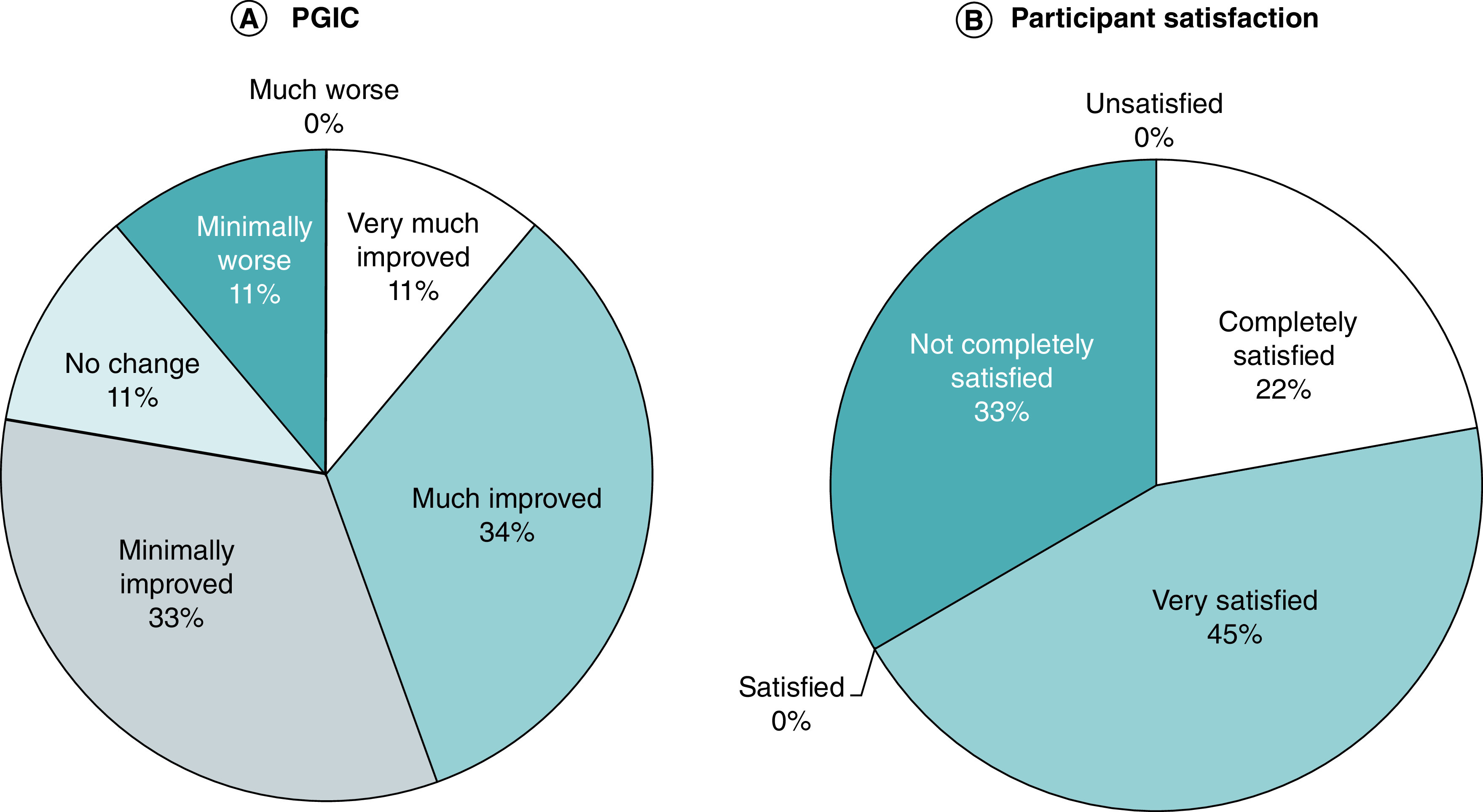
12-month data showing participant satisfaction and patient global impression of change data. **(A)** Categorical PGIC data. **(B)** Participant satisfaction survey. PGIC: Patient global impression of change.

At 12 months, seven participants (78%) recorded improvement using the PGIC, ranging from minimally improved (n = 3; 33%), much improved (n = 3; 33%) and very much improved (n = 1; 11%) (Figure 7A). One participant (11%) recorded no change, and the participant who suffered the work-related injury recorded a slight worsening (n = 1; 11%). For the participants who recorded an improvement (n = 7), a follow-on question was included asking them to stipulate how much they felt they had improved as a result of the therapy. These results varied from 20% to 100% improvement, with a mean improvement of 52%.

Participants were also asked whether they were satisfied with the treatment, to which, 67% (n = 6) were either very satisfied or completely satisfied, and 33% (n = 3) were not completely satisfied (Figure 7B).

### Structural changes within the IVD

One of the secondary objectives of this study was to assess if ADMSC therapy offered signs of disease-modifying potential, in other words, reducing progression of DDD and restoring or improving disc function through regeneration of the IVD. All participants had a baseline MRI, and this was repeated at 12 months.

**Figure 8.**
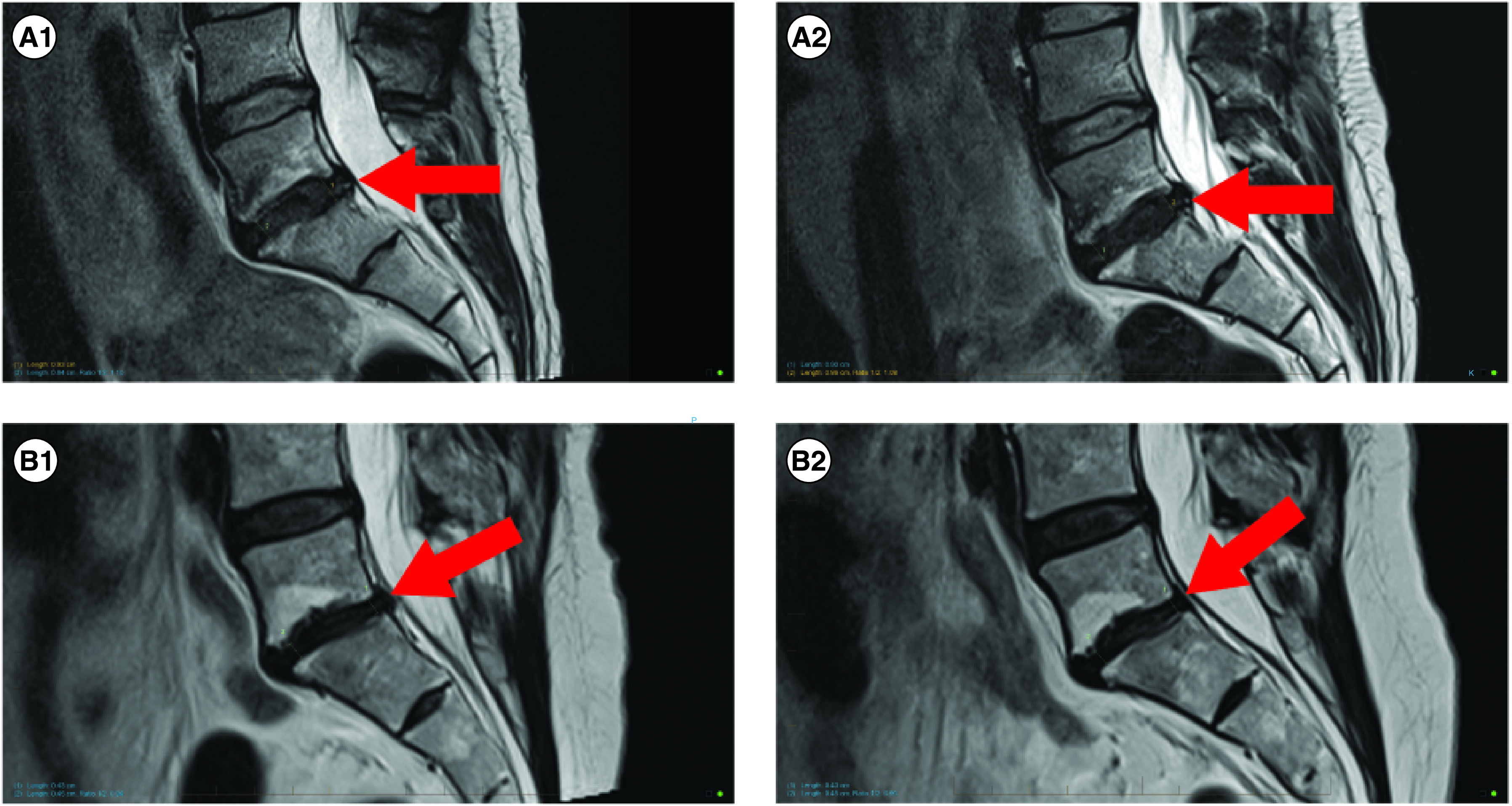
MRI images showing two participants at (i) baseline and (ii) 12 months post procedure. **(A1 & 2)** Represents participant 04 from Table 5 and shows no changes from baseline to follow-up. **(A1)** Participant 04, baseline. **(A2)** Participant 04, 12 months. **(B1 & 2)** Represents participant 07 from Table 5 and shows no changes in disc height, no progression of Modic 2 endplate changes and a reduction in disc protrusion size. **(B1)** Participant 05, baseline. **(B2)** Participant 05, 12 months.

In all nine participants, MRIs taken at 12 months showed no change to disc height and no progression of endplate degenerative changes in any of the nine participants. Annular fissures observed in three participants lessened or resolved and two participants had a reduction in size of their disc bulge/protrusion. Details of each participant's MRIs at baseline and at 12 months follow-up are shown in Table 5, with two representative participants' images shown in Figure 8.

**Table 5.** Baseline and 12-month MRI findings.

Pt no.	Treatment disc	Baseline MRI findings	12–month MRI findings
02	L5/S1	Desiccated disc with shallow disc protrusion Hyperintense posterior peripheral zone (annular fissure) Normal disc height	No changes in disc height No progression of shallow disc protrusion Hyperintense posterior peripheral zone (annular fissure) has resolved
03	L5/S1	Narrowed desiccated disc Mild broad based disc protrusion Endplate cortical irregularity with type 2 Modic changes	No changes in disc height or disc desiccation No change in disc protrusion No change in Modic 2 endplate changes
04	L5/S1	Desiccated disc with moderate to severe narrowing at its right lateral margin. Modic I endplate degenerative change. Disc protrusion, mild disc bulge with small posterior annular tear	No changes in disc height No progression of endplate degenerative changes No change in small posterior annular tear
07	L5/S1	Narrowed desiccated disc Small shallow disc protrusion Modic 2 endplate changes	No changes in disc height No progression of Modic 2 endplate changes Disc protrusion reduced in size
08	L5/S1	Disc desiccation. Normal disc height. Shallow posterior disc protrusion. Hyperintense posterior peripheral zone	No changes in disc height No progression of endplate degenerative changes Stable disc protrusion and disc height Hyperintense posterior peripheral zone (annular fissure) has reduced in size
11	L5/S1	Disc desiccation with normal disc height Small annular fissure Broad based disc protrusion	No changes in disc height No progression of endplate degenerative changes Reduction in size of annular fissure
13	L3/4	Mild disc narrowing posteriorly Disc desiccation with very minor disc protrusion	No changes in disc height or disc protrusion No progression of endplate degenerative changes
14	L2/3	Disc desiccation. Very mild loss of disc height Small shallow disc protrusion. Inferior L2 endplate Schmorl's node with mild type 2 Modic endplate changes	No changes in disc height or disc protrusion No progression of endplate degenerative changes
16	L5/S1	Modic type 2 end plate changes Desiccated disc with moderate reduced height. Circumferential disc bulge	Disc bulge reduced in size Mild reduction in posterior disc space height No progression of endplate degenerative changes

pt: Patient.

### Complications & adverse events

No unexpected or serious adverse events (AEs) were recorded for either the lipoharvest or disc injection procedures. Most participants experienced mild pain (n = 7; 78%), minimal discharge (44%) and/or mild bruising (n = 8; 89%) following the lipoharvest procedure and one participant experienced moderate pain (11%). These AEs resolved in all cases without the need for interventions or an increase in analgesia and are considered to be expected AEs. No infections at puncture sites were recorded, and healing was normal in all cases.

One expected adverse event related to the disc injection was noted during the study period – a pain flare that required opioid analgesia. This flare had resolved by the 1-month follow-up visit.

## Discussion

Previous studies on the use of MSCs for disc degeneration have shown benefits for the treatment of discogenic LBP, both in reducing pain and assisting in disc regeneration [37–40] with these past studies using a variety of cellular therapies. In a fresh approach, this study aimed to investigate the use of autologous ADMSCs at low concentrations, due to their relative ease of harvest, potential for extended proliferation and supportive safety profile.

The demographics of the group were favorable, with average BMI (25.4) bordering the healthy range, isolated modic changes and other causes of pain (such as facet and sacroiliac joint pain) excluded following assessment in an interventional pain clinic. In addition, many of the participants were still able to work, and the participants did not have any known psychological problems that would interfere with the study outcomes.

Importantly this trial indicated intradiscal ADMSC therapy to be safe with treatment well tolerated. No unexpected or serious adverse events were recorded. Whilst a single participant experienced a flare post therapy this responded to use of appropriate analgesia and was self-limiting.

The overall results shown in this pilot study are positive and show benefits in relation to both pain and functional outcomes over 12 months of follow-up for participants with single-level lumbar disc degeneration.

Using the NPRS, the majority of participants reported improvements in both their average (78%; n = 7) and most severe (67%; n = 6) pains. Changes in percentage scores by 30% or more reflect clinically meaningful change [69], and this was achieved in 67% (average pain) and 44% (most severe pain) of participants. In our clinical practice, ≥50% improvement is often viewed as the marker of success, which was achieved for 55% (n = 5) and 30% (n = 3) of participants at 12 months for average pain and most severe pain, respectively.

The functional outcomes data unveiled encouraging quality of life benefits over the 12 months for most participants. While all participants noted problems with performing their usual activities at baseline, most participants (66%; n = 6) no longer had such problems after 12 months. In addition, one participant who was previously unable to perform the usual activities was able to perform them after 12 months. Similarly, all participants recording problems with self-care at baseline reported no problems with self-care after 12 months. Across all the EQ-5D-3L categories, participants who did not record an improvement also did not record any worsening related to the treatment.

The ODI results were also encouraging. All but one participant demonstrated an improvement in functional outcomes, and most participants exhibited substantial improvement. It has long been considered a challenge to place a value for the minimal clinically important difference (MCID) in the ODI [70] and the literature has varied with proposed MCID values [71–73]. We have based our interpretation of clinically meaningful ODI results on a recent publication who proposed a MCID in ODI of ≥30% reduction based on their results of a study involving 23,280 participants [69]. In our study, 6 (67%) participants recorded a change of ≥30% and for five of these, the improvements lead to a change in clinical category as interpreted by the ODI.

Very little can be concluded from the DASS21 results. The stress and anxiety outcomes did not vary much from baseline values in any of the participants. The depression outcomes varied more, with a mixture of improvements and deterioration seen.

The results of the PGIC and participant satisfaction survey were also promising, together with the functional outcomes showing that although the participants were not pain-free, they had an improved quality of life, and most (67%) were either very satisfied or completely satisfied with the treatment outcomes.

Several environmental factors may have indirectly contributed to the improvements seen in the reported quality of life outcome measures. Many participants (n = 5; 56%) reported an increased capacity to work, which may not only affect the specific questions of the surveys but the overall well-being and feeling of worthiness of those individuals. Of the seven participants taking analgesic medication at baseline to treat their pain, three (43%) had also reduced their analgesic medication, which can also have a positive outcome with quality of life if it results in fewer side effects and/or feeling of greater empowerment. Pain reduction may often also lead to an increase in activities, which can improve quality of life for participants. The extent of the undertaking of activities was not explicitly tracked in this study. However, the outcomes of the sitting, walking and standing tolerances and relevant functional measures were predominantly positive in this regard.

In a previous study [52], the use of a higher dose intradiscal injection of MSCs did not see an increase in efficacy between 20 and 40 million cells in participants treated with ADMSCs in HA carrier for discogenic pain. Here, we have been encouraged by promising results in some participants with just 10 million cells in isotonic solution, suggesting that this even lower dose may also be sufficiently efficacious in specific patient populations without co-administration of HA. An effective lower dose is a positive outcome as it holds the potential to deliver efficacious treatment to patients in a faster timeframe. In our study, little can be concluded as to whether a doubling of dose via a second administration 6 months after the first administration would lead to improved efficacy due to high variability in results within a small cohort. We did observe, however, that if participants did not achieve ≥50% improvement in their NPRS results after 6 months, they did not achieve >50% pain improvement after 12 months regardless of whether they received additional dosing. Similar studies have observed the greatest clinical benefit within the first 6 months of stem cell treatment, with only moderate improvement up to 12 months [38,52]. From a clinical perspective, therefore, 6 months may represent a useful timeframe to assess patient responses and determine whether to progress with a different treatment option.

It has long been debated whether MRI provides conclusive evidence concerning the relationship between disc degeneration and the symptoms of chronic low back pain symptoms [74,75]. As mentioned earlier, degenerative rates of between 3 and 15%/year (18–20) would normally be expected, however, in our study, the MRI results showed stability from baseline in all nine participants. No changes to disc height or progression of endplate degenerative changes were observed. Annular fissures observed in three participants lessened or resolved, and two participants had a reduction in size of their disc bulge/protrusion. These morphological improvements may have been due to the treatment or to natural history [76] and it is interesting to consider the consistency in degradation stabilization in this cohort despite variability in the pain and functional outcomes. Improvements in pain and function outcomes without change to disc height at 12 months has also been reported previously following IVD treatment with autologous bone marrow-derived MSCs [38] with this study observing an increase in fluid content of the discs, which was not assessed here, a potential limitation of this study.

As discussed above, there are several treatment options available for treating LBP associated with DDD, such as pain medication, physical exercises, interventional approaches, multidisciplinary biopsychosocial rehabilitation and surgery [74,75]. Medication can be beneficial in some instances, however, the use of pain medications shows most benefits in acute pain and their use in chronic pain often does not provide an appropriate solution, offering approximately 30% pain relief together with significant adverse events [77]. Furthermore, the long-term use of opioids – the most commonly prescribed drugs used to treat chronic LBP [75] – can cause greater problems with dependence and other adverse consequences, contributing to the current opioid epidemic [75,77].

Exercise therapy is favorable in terms of being a conservative option which has shown some promise with decreasing pain and improving function, however, it has only shown modest benefits and typically does not result in clinically significant improvements [74]. Psychological interventions are often encouraged but are typically advocated in conjunction with physical treatments in a multibiopsychosocial approach. This has shown promise but also has limited evidence reliant on the physical treatment selected and continues to have mixed opinions in clinical practice [75].

Interventional therapies have shown mixed results but clear positive outcomes have been observed with epidural injections in patients presenting with axial LBP [75]. A disadvantage with these treatments is that the treatment can wear off and additional injections are often required [78]. Manchikanti *et al. *reported an improvement in NPRS and ODI scores after 12 months in patients treated with epidural injection alone (group 1: NPRS 63% improvement, ODI 56% improvement) and epidural injection together with steroidal injection (group 2: NPRS 72% improvement, ODI 72% improvement), both groups requiring approximately four procedures over the same time interval [79].

In clinical practice, surgical treatments are also used to treat patients presenting with DDD. While some studies have shown benefit with such surgeries, other studies have shown no difference in disability and functional outcomes between surgical fusions and disc replacement compared to conservative therapies [77], and others have shown benefits only in the more extreme cases of disc degeneration [74]. Clinical follow-up has also revealed ongoing problems in many patients treated by surgical means, such as fusion failure (10% requiring reoperation typically within 5 years), chronic pain and adjacent segment degeneration following posterior lumbar interbody fusion [80]. Surgical intervention has indeed been criticized for its lack of evidence [77] and is viewed by many to only be considered as the last treatment option after less invasive options have already been exhausted [75] and/or recommended only for a minority of patients [74].

Stem cell therapy to treat DDD has shown promise in other early studies [81] and the results we see here are similarly reflected. Given the versatile literature and results reported across all DDD treatments, it is difficult to compare the techniques without a very carefully designed randomized clinical trial. Even then, there will undoubtedly be complexities such as with blinding. Initial results of this study are encouraging, indicating that stem cell therapy may provide a very useful and attractive treatment option for patients suffering from DDD.

The three top responders in this study based on both average and most severe NPRS results presented with Modic type 2 changes at baseline, and no annular tears. The one other participant who presented with these characteristics was the participant who suffered a work-related injury at 12 months and therefore recorded poor outcomes at 12 months. Only one participant presented with Modic type I change, together with an annular tear, who demonstrated poor outcomes with no improvement in severe pain and minor (14.3%) improvement in average pain at 12 months. Annular tears were generally seen as negative indicators, although one participant reached 50% improvement at 12 months in most severe and average pain scores following a second injection. Whilst little can be implied from such small cohorts, these outcomes warrant further investigation into low dose autologous ADMSC treatment in a more select patient population with focus on patients with Modic type 2 changes.

Study limitations of this trial include a lack of control group and a small sample size. The trial was directed toward patients suffering from DDD and excluded participants with radicular type pain, a disc height <50%, or exhibiting multiple levels of disc degeneration. While no significant conclusions can be drawn from the data presented, the use of low ADMSCs concentration injections for disc degeneration in this small sample was shown to be a safe, well tolerated procedure with minimal morbidity and mostly positive outcomes.

## Conclusion

LBP is a leading cause of disability in our society and affects a significant proportion of the population. Current conservative therapies are limited, and conventional treatments such as surgery have shown inconsistent improvements and may have unwanted side effects.

In this study, autologous ADMSCs in isotonic solution resulted in pain and functional improvements for most study participants over 12 months of follow-up. 78% of participants reported an overall improvement and 67% were very satisfied or completely satisfied with the treatment. MRI analysis after 12 months indicated no change to disc height, no progression of endplate degenerative changes and (partial) resolution of annular fissures. Participants with Modic type 2 changes and no annular tears appeared to benefit most from this treatment.

Additionally, ADMSC therapy was well-tolerated, had no safety issues, and no treatment-related serious adverse events. Coupled with the positive outcomes in pain and functional improvements, this treatment shows potential for helping people suffering from debilitating discogenic LBP.

## Future perspective

Regenerative cellular therapies form part of accepted medical practice in the areas including bone marrow and tissue transplantation, blood transfusion and *in* *vitro* fertilization. More recent advances in our understanding of the pathology of musculoskeletal complaints in addition to the properties of mesenchymal stem cells and their ability to influence healing through paracrine and cell-to-cell interaction have seen the emergence of additional and promising regenerative medicinal pathways. Discogenic LBP is a condition associated with significant disability with poor outcome associated with conventional therapies including physiotherapy and surgical intervention. Improvements in pain and quality of life measures in combination with observed structural improvements indicates that ADMSCs is a promising future regenerative cellular therapy in the treatment of LBP. Further studies are needed over a larger scale to further investigate some of the outcomes observed in this study. In particular, a focus of participants presenting with Modic type 2 changes and no annular tears could confirm whether this particular cohort are more suited to this treatment option, as this early data suggests.

**Summary points**

- Low back pain (LBP) is a major health problem, affecting approximately 60–80% of the adult population at some stage in their life. It is the second most common reason for physician visits and work disability and is associated with substantial health care costs and work absenteeism.
- The intervertebral disc is a common source of LBP, being the prime source in about 40% of complex chronic LBP presentations.
- Past treatment options have centered primarily around pharmacological and/or surgical interventions with limited evidence of success. Surgical interventions such as discectomy and fusion often yield uncompelling outcomes and may come with significant morbidity.
- Early pre-clinical and clinical trials have indicated the ability of intra-discal mesenchymal stem cell therapies to improve pain and function.
- In this feasibility study the use of low dose autologous adipose-derived mesenchymal stem cells (ADMSC)s in isotonic solution resulted in pain and functional improvement with improvement in quality of life as measured by validated outcome measures.
- The majority of participants reported improvements in both their average and most severe pains. Two-thirds of participants achieved clinically meaningful change in pain (>30% improvement in pain scores).
- 78% of participants reported an overall improvement and 67% were very satisfied or completely satisfied with the treatment.
- MRIs demonstrated no changes to disc height, no progression of endplate degenerative changes and (partial) resolution of annular fissures.
- ADMSC therapy was well tolerated with no related serious adverse events.
- Autologous ADMSC intra-discal therapy represents an exciting advancement in the treatment of disc related LBP.
